# Logic circuit prototypes for three-terminal magnetic tunnel junctions with mobile domain walls

**DOI:** 10.1038/ncomms10275

**Published:** 2016-01-12

**Authors:** J. A. Currivan-Incorvia, S. Siddiqui, S. Dutta, E. R. Evarts, J. Zhang, D. Bono, C. A. Ross, M. A. Baldo

**Affiliations:** 1 Department of Electrical Engineering and Computer Science, Massachusetts Institute of Technology, Cambridge, Massachusetts 02139, USA; 2 Department of Physics, Harvard University, Cambridge, Massachusetts 02138, USA; 3 Physical Measurement Laboratory, National Institute of Standards and Technology, Boulder, Colorado 80305, USA; 4 Department of Materials Science and Engineering, Massachusetts Institute of Technology, Cambridge, Massachusetts 02139, USA

## Abstract

Spintronic computing promises superior energy efficiency and nonvolatility compared to conventional field-effect transistor logic. But, it has proven difficult to realize spintronic circuits with a versatile, scalable device design that is adaptable to emerging material physics. Here we present prototypes of a logic device that encode information in the position of a magnetic domain wall in a ferromagnetic wire. We show that a single three-terminal device can perform inverter and buffer operations. We demonstrate one device can drive two subsequent gates and logic propagation in a circuit of three inverters. This prototype demonstration shows that magnetic domain wall logic devices have the necessary characteristics for future computing, including nonlinearity, gain, cascadability, and room temperature operation.

## Abstract (web summary)

Ferromagnetic nanowires act as conduits for magnetic domain walls which may in principle be used to encode and propagate information. Here, the authors present current-based nanowire domain wall logic prototypes with operational properties required for real devices.

## Graphical abstract

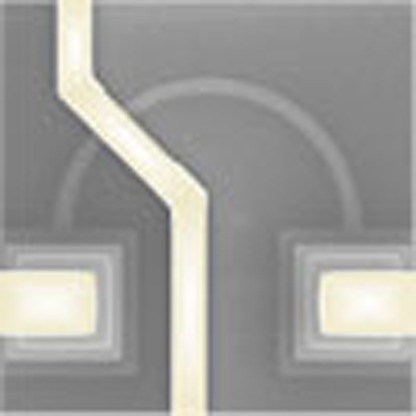

There is great interest in encoding information in magnetic materials for future memory and logic^1^,^2^,^3^,^4^,^5^,^6^. Recent developments in perpendicular anisotropy materials^7^,^8^ and spin Hall effect-assisted domain wall (DW) switching^9^,^10^,^11^ have reduced the current density required to switch a magnet. Many groups are working on instantiations of magnetic logic and memory, for example, ratchet memory^12^, three-terminal magnetic random access memory^13^, majority logic gates^14^,^15^, nanomagnetic logic^16^ and mLogic^17^. These devices show promise for future computing, and work has been done to simulate and build them. However, so far, little work has shown experimentally verified DW-based devices that can perform logic, be used in circuits, and have a gate-like basic element that can be used to build more complex circuits.

In this work, we present magnetic device prototypes that are capable of integration in logic circuits and easily adaptable to the continuing advances in spintronics materials and scaling. The device is a memory cell that we adapt to logic. Information is stored in the position of a DW in a short, narrow ferromagnetic wire, written using spin-torque transfer^18^ from pulsed voltage clocks, and read out using a magnetic tunnel junction (MTJ)^19^. The device is nonvolatile and is predicted to scale to switching energies competitive with field-effect transistors^1^. We demonstrate that a single device can perform buffer and inverter operations, one device can drive two subsequent devices and three devices in series act as a circuit of three inverters. These results provide a path for realizing spintronic circuits.

## Results

### Domain wall logic device structure

**Figure 1:**
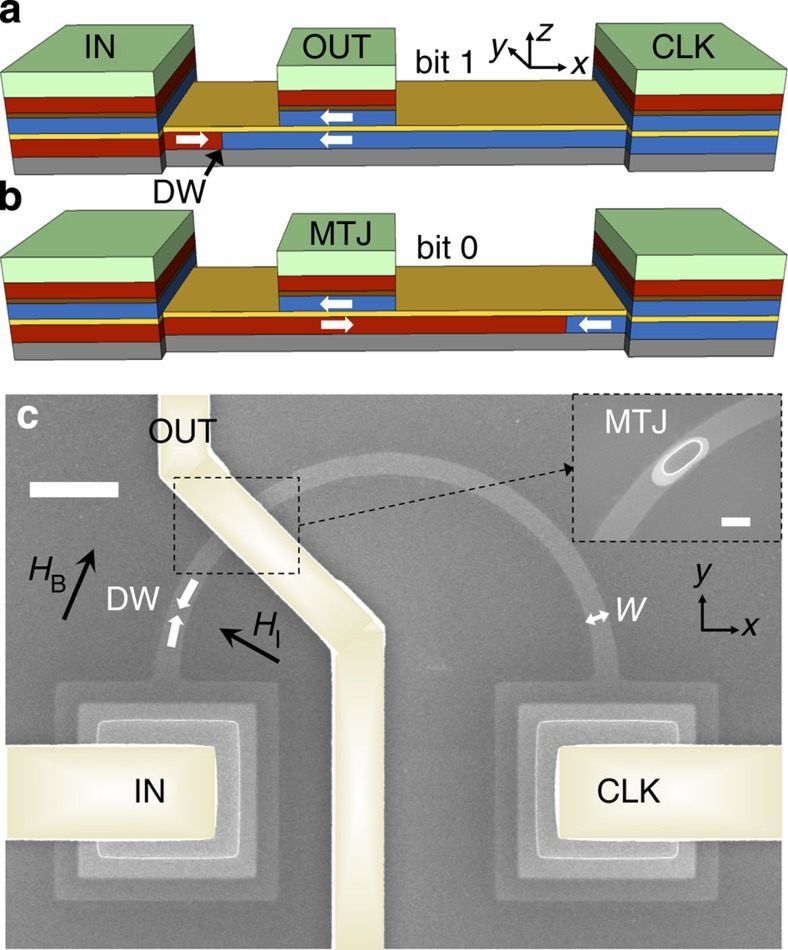
Logic device cartoon and SEM. (**a**), Cartoon of the domain wall (DW) logic device, comprised of a magnetic wire with a DW and a magnetic tunnel junction (MTJ) to read out the DW position. Right-pointing magnetic moments are red and left-pointing are blue (grey represents Ta, brown Ru, and light green IrMn). Magnetization direction is also shown by the white arrows. The MgO tunnel barrier is shown in yellow. The device has three terminals, IN, CLK, and OUT. When the wire magnetization is parallel to the MTJ magnetization, the current at the OUT terminal is high, representing bit 1. (**b**), Cartoon showing the DW switched to the opposite side of the device, representing bit 0. (**c**), SEM image of a DW-Logic device prototype, showing the magnetic wire of width *w*=400 nm and the MTJ under the center electrode with area 1 μm × 400 nm, also shown in the inset. Electrodes are coloured in light gold. The initial DW position is shown by the white arrows. The black arrows show the orientation of the initialization field **H**_I_ and bias field **H**_B_. Scale bars, 2 μm (**c**), 400 nm (**c**, inset).

The prototype is shown schematically in Fig. 1a,b. It consists of two parts: a ferromagnetic wire containing a DW and a MTJ. The wire is composed of sputter-deposited thin films on a Si substrate: SiO_2_ (200 nm)/Ta (3 nm)/Co_40_Fe_40_B_20_ (*t*=4 nm)/MgO (1 nm). The CoFeB has in-plane magnetic anisotropy; while perpendicular anisotropy will be more energy-efficient in scaled devices^1^,^20^, as explored in ref. 1, for initial prototypes the logic behaviour is independent of anisotropy type. The MTJ stack is CoFeB (2.5 nm)/Ru (0.8 nm)/CoFeB (2.5 nm)/Ir_20_Mn_80_ (10 nm)/Ta (3 nm)/Ru (7 nm). There are three terminals: IN, CLK and OUT.

Figure 1c shows a scanning electron microscope (SEM) image of a fabricated device. The wire width is *w*=400 nm and is shaped into a curve to allow initialization of the DW using a globally applied field **H**_**I**_. Micromagnetic simulations predict that the DW is transverse, see Supplementary Fig. 1. The MTJ sits under the center electrode. The IN and CLK terminal pads are 5 μm × 5 μm MTJs used to contact the magnetic wire, much larger than the center MTJ. At this size there is negligible magnetoresistance in the pads due to pinholes in the MgO layer, so their tunnel junctions are ignored. The estimated series resistance of the pads is about 100 Ω, small compared with the wire resistance.

Device operation includes an initialization step, a write step and a read step. First, *μ*_0_*H*_I_=200 mT is applied to initialize the DW on the left, where *μ*_0_ is the permeability of free space. This field is large enough to saturate the magnetic moments in 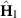, such that when the field is removed a DW is nucleated along the field direction. By having a curved wire, we can set the initial DW position by choosing the field angle. The magnetization is parallel on either side of the MTJ, and its resistance is low (bit 1, Fig. 1a). See Supplementary Figs 2 and 3 for field-driven device characteristics. For current-driven behaviour, a 1-μs voltage pulse is applied to the IN terminal with CLK grounded. It is theoretically possible to use a three-phase sinusoidal clock to save energy^1^,^21^. If the input current, *I*_IN_, is above the threshold current of the DW, *I*_T_, then the current can translate the DW past the MTJ using current-induced DW motion^22^, switching the MTJ to a high-resistance state (bit 0, Fig. 1b). Most likely, the current-induced switching is a combination of spin transfer torque and heat-assisted depinning^23^. *I*_T_ depends on both intrinsic and extrinsic pinning of the DW from sources such as line edge roughness^24^ and magnetocrystalline defects.

The device can then be read by a voltage pulse applied to CLK sunk to the input of the next device. The current at the output terminal, *I*_OUT_, will be high or low depending on the position of the DW, and can be fed into the next device stage. To avoid damage to the devices at high current density, DW motion is assisted by a global bias field **H**_B_, aligned parallel to the initial DW position. The bias field could be eliminated by using a magnetic material with lower *I*_T_, or by scaling the devices to smaller length scales. Simulations and experiments suggest that the current density required to depin a DW decreases with wire width to sub-50 nm sizes^1^,^22^.

### Single device behaviour as inverter and buffer gates

**Figure 2:**
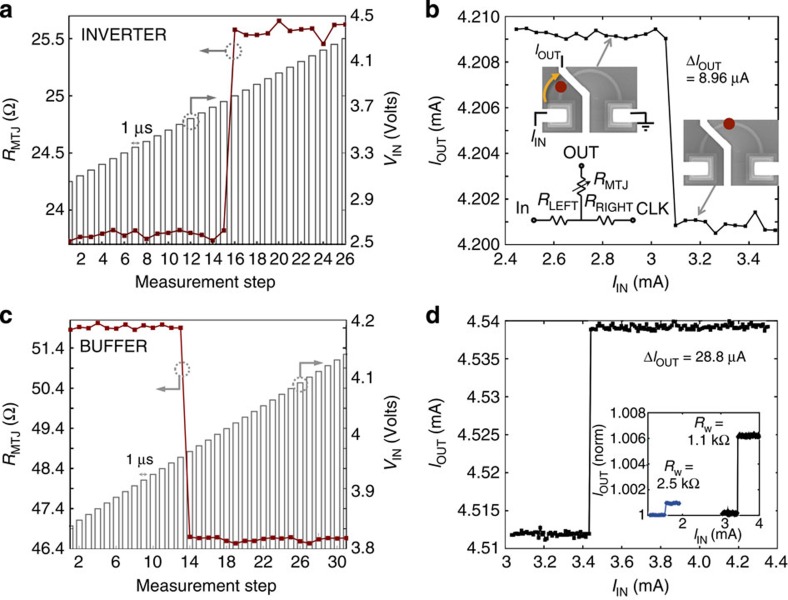
Inverter and buffer operation. (**a**), Transient showing the MTJ resistance and applied voltage pulses between IN and CLK versus time. At 3.80 V, the device switches from low resistance (bit 1) to high resistance (bit 0). (**b**), Transfer characteristic of *I*_OUT_ through the MTJ versus *I*_IN_ across the wire. The SEM images show the configuration below *I*_T_, with the DW position approximated by the red dot and *I*_IN_ electron flow direction shown by the yellow arrow, and the approximate DW position after *I*_T_. inset, Simple circuit diagram to represent the device, including the wire resistance and the variable tunnel junction resistance. (**c**), Transient behaviour showing a buffer operation, plotting *R*_MTJ_ and *V*_IN_ versus measurement step as we increase the 1-μs voltage pulse amplitude in 0.01 V steps. This device has similar *R*_w_ to the previous device, but slightly higher *R*_MTJ_ and *TMR*. At 3.97 V, the device switches from a high-resistance output to a low-resistance output. (**d**), Transfer characteristic for the buffer. inset, Comparison of this transfer characteristic (right black curve) to that of another device (left blue curve) with less well matched *R*_w_ and *R*_MTJ_.

A single device can be used as a logic gate when the current at the input terminal, *I*_IN_, is the sum of multiple input currents. Figure 2a shows an example of a device acting as an inverter, which can be used to do a two-input NAND operation, with parallel MTJ resistance *R*_P_=23.7 Ω, antiparallel MTJ resistance *R*_AP_=25.7 Ω, wire resistance *R*_w_=1.18 kΩ and tunnel magnetoresistance *TMR*=(*R*_AP_−*R*_P_)/*R*_P_ × 100=8.4% (ref. 25). Initialization with **H**_I_ sets the MTJ initially in a parallel state (bit 1), with the DW on the left as in Fig. 1a,c. A bias field *μ*_0_*H*_B_ =3.0 mT is then applied parallel to the DW. We apply a series of 1-μs voltage pulses, *V*_IN_, increasing in 0.05 V steps, to the IN terminal. Each input pulse is followed by a 100 mV d.c. voltage applied to the CLK terminal to measure the resistance through the MTJ, *R*_MTJ_. Between *V*_IN_=3.75 V to 3.80 V, corresponding to an input current of *I*_T_=3.099 mA±0.041 mA (with range determined by the voltage step) and current density *J*_T_=1.9 × 10^12^ A m^−2^, the DW is translated across the MTJ, switching the output from *R*_P_ (high current, bit 1) to *R*_AP_ (low current, bit 0).

The current is calculated using the circuit in Fig. 2b (inset), with the wire resistance represented by *R*_w_=*R*_LEFT_+*R*_RIGHT_ and the MTJ represented by a variable resistor *R*_MTJ_. For an isolated device, we define the input current as


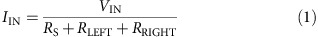


where *R*_S_ is the load resistor from the voltage supply. If we read each device using *V*_OUT_, then we define the output current for an isolated device as


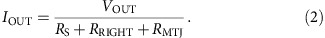


Device stages are pulsed in sequence, where in each stage a device can also have an input resistor that is tuned to put the input current in the correct range to read the data from the previous stage. Thus, if the input current originates from two logic gates, with output resistances such that the input currents sum in the subsequent gate, then that device switches from 1 to 0 only when the input current exceeds *I*_T_. The resulting transfer characteristic is in Fig. 2b. The change in the MTJ resistance modulates the output current by Δ*I*_OUT_=8.96 μA, assuming a CLK voltage pulse of 3.80 V and no external load attached to the OUT terminal.

Figure 2c shows a device instead acting as a buffer gate. The buffer is shown for a different device: *R*_P_=46.5 Ω, *R*_AP_=52.1 Ω, *R*_w_=1.10 kΩ, and *TMR*=12%. The device is initialized the same as previously. We program it to act as a buffer rather than an inverter by applying a higher bias field *μ*_0_*H*_B_=4.8 mT, above the field switch of the reference layer of the MTJ but below that of the DW, initializing the MTJ in the antiparallel state (bit 0). The DW switches across the MTJ between 3.96 and 3.97 V, or *I*_T_=3.441 mA±0.009 mA, *J*_T_=2.0 × 10^12^ A m^−2^. The output switches from high resistance (low current, bit 0) to low resistance (high current, bit 1). Across multiple devices, the transfer characteristic shows a very sharp switch between resistance states, Δ*V*_IN_<0.01 V, the limit of our voltage supply. This enables stable 0 and 1 outputs even with *TMR*=8–20% seen in our devices. At the switch, Δ*I*_OUT_/Δ*I*_IN_=3.1. In Fig. 2d we plot *I*_OUT_ versus *I*_IN_ for the isolated buffer device with *V*_OUT_=3.97 V. The change in current between the 0 and 1 output bits is Δ*I*_OUT_=28.8 μA assuming no output load.

While Δ*I*_OUT_=28.8 μA is an appreciable difference in current for stable 0 and 1 bits, the fractional output current change is Δ*I/I*=(*I*_P_−*I*_AP_)/*I*_AP_=0.3% of the bit 0 output current *I*_AP_,_OUT_=4.5 mA. In an ideal device, Δ*I/I* should be maximized for the best noise margin and potential fanout. Using Equation 2 with fixed *V*_OUT_, and assuming that the gate drives a single subsequent ferromagnetic wire, we find


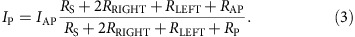


If we define the output resistance in the parallel state *R*_OUT_=*R*_P_+*R*_RIGHT_ and the load resistance *R*_LOAD_=*R*_LEFT_+*R*_RIGHT_+*R*_S_, then we can define a figure of merit


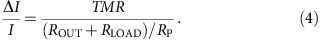


Equation 4 shows that the highest noise margin and fanout is obtained by maximizing the *TMR* and matching the MTJ parallel resistance and the resistance of the wire.

Figure 2d (inset) compares *I*_OUT_ versus *I*_IN_ of the original buffer device (black curve) to another tested device with higher *R*_w_=2.54 kΩ, *R*_P_=12.4 Ω, and *TMR*=21% (blue curve). Even though the *TMR* is higher, since 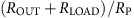 is also higher we find lower Δ*I/I*=0.1%. For a given wire resistivity and MTJ resistance-area product, we can reduce the ratio between the wire and MTJ resistances by properly choosing the size of the wire and the size of the MTJ. Future devices with size optimized for resistance instead of fabrication constraints are expected to exhibit a substantially larger percent change in current. Increasing the MTJ quality to *TMR*=100–600% (ref. 5) and choosing the wire and MTJ geometries to match 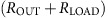 and *R*_P_ would increase Δ*I/I* up to 100–600%, and implementing PMA and spin Hall-induced switching could reduce power consumption and *J*_T_ by 100 × (ref. 26).

### Device variability and reversibility

**Figure 3:**
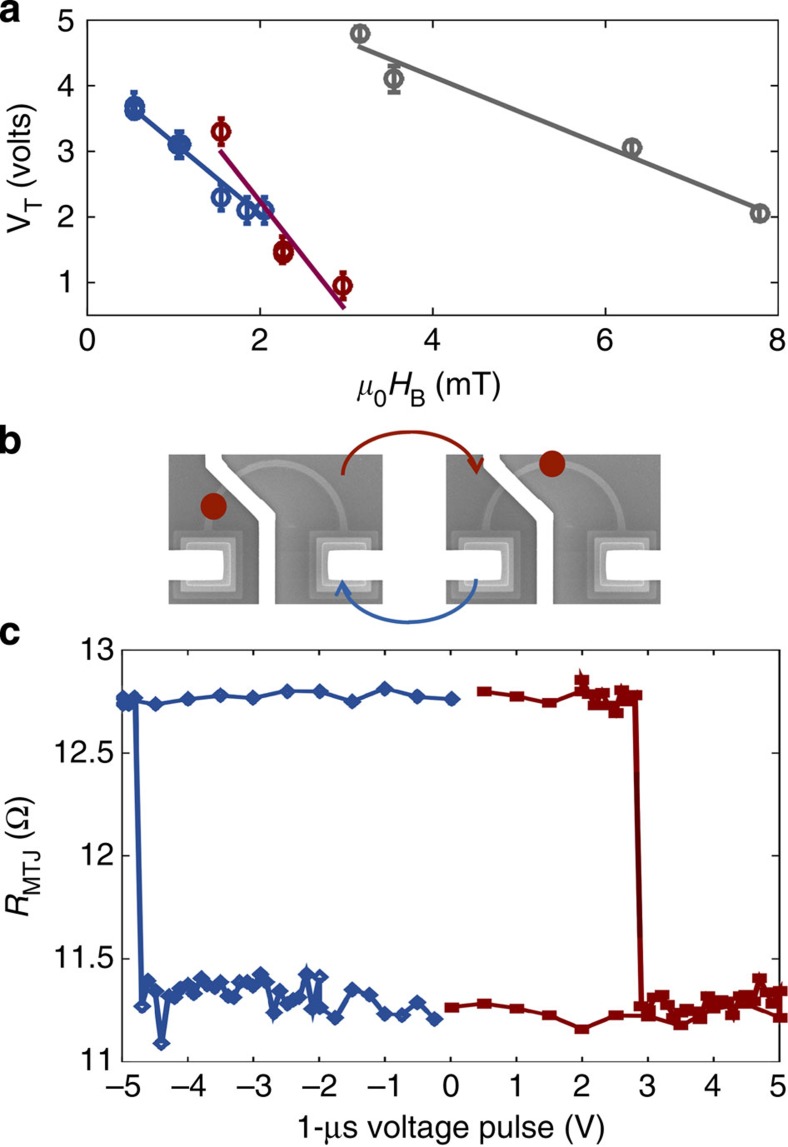
Variability and reversibility. (**a**) Threshold voltage versus bias field for three different devices, where the error bars represent the depinning voltage standard deviation repeated at least 3 × , with the DW reinitialized between each. Grey=device 1, red=device 2 and blue=device 3. The lines are a linear fit. Devices 2 and 3 were fabricated together, while device 1 was fabricated at a later time on a different wafer piece. (**b**) SEM images showing reversibility setup, with the DW moving back and forth past the MTJ as shown by the red dots. (**c**) *R*_MTJ_ versus pulsed *V* showing reversibility of device 1's state. In the positive direction *μ*_0_*H*_B_=3.3 mT; in the negative direction *μ*_0_*H*_B_=−6.2 mT. The red dot in the SEM images is the approximate DW position at high and low *R*_MTJ_, respectively.

In Fig. 3a we plot the threshold voltage, *V*_T_, versus *H*_B_ for three different devices to assess the variability in *V*_T_=*I*_T_(*R*_w_) and between devices. The DW depinning voltage is repeatable within ±(0.15±0.1) V at each bias field. This is without externally fabricated pinning, such as notches^27^, that can supply more repeatable depinning at the expense of higher depinning currents. There is a linear trend within a device, but variation between different devices. Devices 2 and 3 were fabricated at the same time from the same 10 mm × 10 mm thin-film wafer piece, while device 1 was fabricated at a different time from a separate wafer piece and shows higher device-to-device variation in the *V*_T_ versus *H*_B_ slope. Thus, we can conclude that the device-to-device variation seen in the prototypes arises from variations in fabrication, for example, electron-beam resist age and etching rates, and variation in the MgO thickness across the initial 3-inch diameter thin film wafer, which causes variation in *TMR* across the wafer.

We can estimate the zero-field current density from the linear trends: device 1 *V*_T_ (*H*_B_=0)=6.27 V. Converting to *I* with *R*_w_=4.4 kΩ, we find *I*_T_ (*H*_B_=0)=1.4 mA and *J*_T_ (*H*_B_=0)=8.75 × 10^11^ A m^−2^. This is in agreement with the current density needed to translate a DW in in-plane magnetic anisotropy wires^20^.

Figure 3b shows the reversibility behaviour of device 1. After initialization in the buffer configuration, at +2.9 V *R*_MTJ_ switches low; reversing 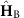 we see *R*_MTJ_ switch high again at −4.8 V. *H*_B_ is higher on the negative side of the loop since the field direction is less parallel to the DW.

### Concatenation behaviour of multiple devices in circuits

**Figure 4:**
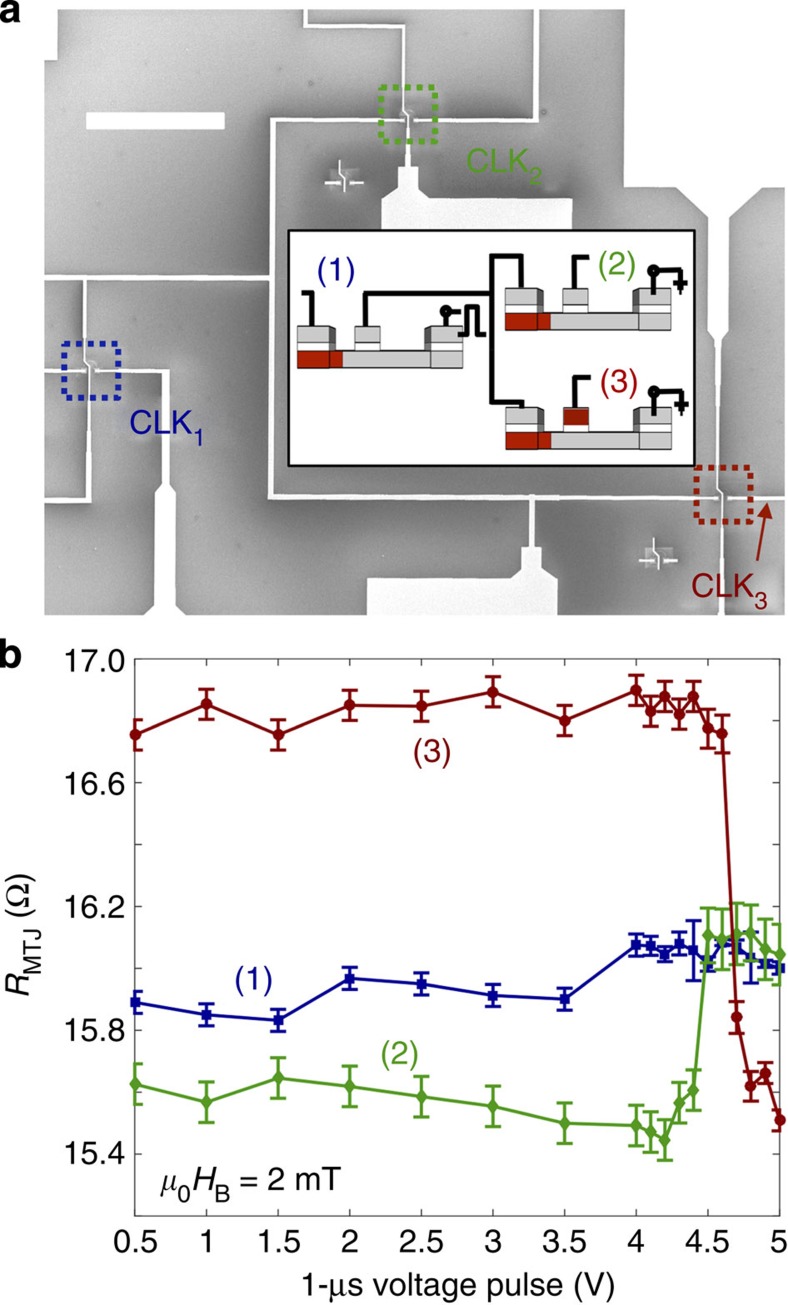
Concatenation. (**a**) SEM image of one device output connecting to the inputs of two devices. Voltage pulses are applied to CLK_1_ with CLK_2_ and CLK_3_ grounded. Scale bar, 100 μm. (inset) Cartoon of the initial configurations of the three devices, with the DWs initialized on the left. Red signifies magnetized right and grey signifies magnetized left. (**b**), Plot of MTJ resistance versus pulsed voltage applied at CLK_1_, showing one device can power a switch in two subsequent devices. Here, the error bars represent the average noise fluctuation in *R* monitored for each device at each voltage step.

Figure 4a is a SEM image showing three devices concatenated together, with device 1's output feeding the input of device 2 and device 3. The dotted boxes identify devices shown in Fig. 1c. The initial state is conceptualized in Fig. 4a (inset). After initialization and *μ*_0_*H*_B_=2.0 mT, devices 1 and 2 are in an inverter configuration, and device 3 is a buffer. We then apply 1 μs voltage pulses to the CLK_1_ terminal with the CLK_2_/CLK_3_ terminals maintained at ground and all other terminals floating. Figure 4b shows *R*_MTJ_ after each pulse. The output current from device 1 switches both device 2 and device 3, at 4.5 V±0.3 V and 4.7 V±0.2 V, respectively (range is from three experiment repeats). The resistance of device 1 remains unchanged because its DW is initialized on the left side of its wire, and thus we are able to read device 1 without disturbing its logic state. This demonstrates that one gate can drive two gates, and that we can read the state of device 1 and then use that output current as the input to write devices 2 and 3. We can power more than two gates by further increasing the voltage pulse amplitude or tuning the resistance-area product of the MTJ to output higher current.

**Figure 5:**
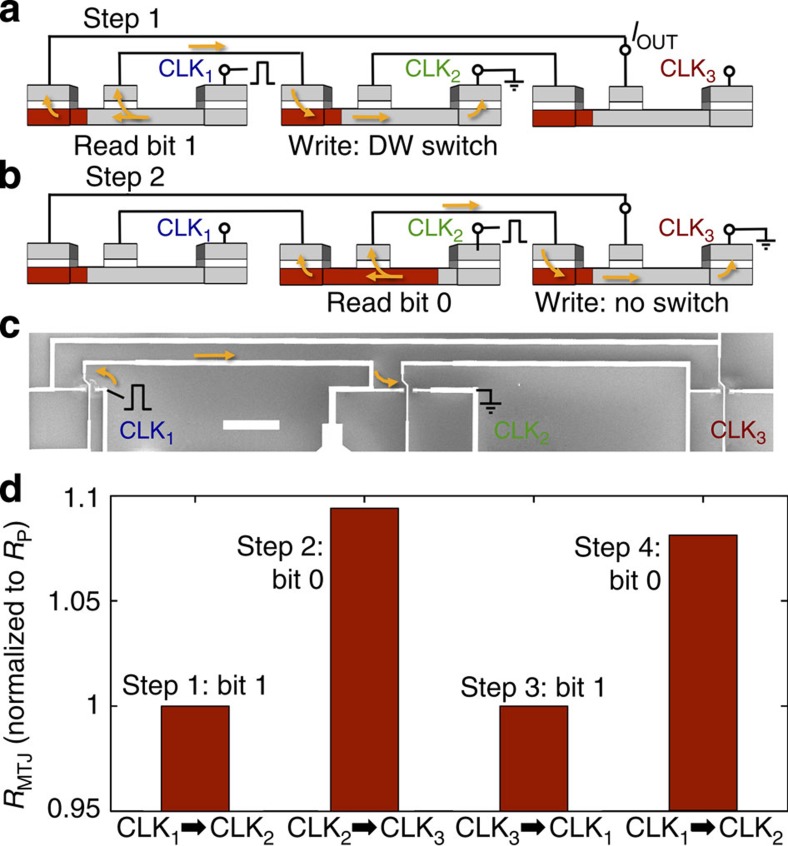
Three inverters circuit. (**a**) Cartoon showing three devices connected in series, with the output of device 3 feeding back into the input of device 1. The devices are initialized with their domain walls (DWs) on the left and the tunnel junctions parallel. In the experiment, at Step 1 we apply a voltage pulse at CLK_1_ to read the state of device 1 while writing the state of device 2. The voltage pulse is applied between CLK_n_ and CLK_n+1_. Yellow arrows represent electron flow direction. In this case the DW in device 2 switches. (**b**) Cartoon of Step 2, where we now apply a voltage pulse at CLK_2_ to read the state of device 2 while writing the state of device 3. In this case the DW in device 3 does not switch. In Step 3 and Step 4 this is repeated at CLK_3_ and CLK_1_, respectively. (**c**) SEM of three devices in series. *R*_P1_=23.8 Ω, *R*_P2_=19.0 Ω, and *R*_P3_=18.4 Ω. Scale bar, 50 μm. (**d**) Plot of the MTJ resistance at each step in the three inverters circuit, showing the oscillation of the DW position at each step between the parallel (low MTJ resistance, bit 1) and antiparallel (high MTJ resistance, bit 0) device states.

Figure 5 shows a circuit of three inverters that concatenates three devices in series. The output of device 3 is fed back into the input of device 1. We initialize all devices in the inverter configuration, depicted in Fig. 5a. To drive the logic flow, we apply sequential voltage pulses to CLK_1_, CLK_2_ and then CLK_3_. For example, in Step 1, the voltage pulse applied to CLK_1_ with CLK_2_ grounded allows us to read device 1 while writing device 2. Since the output of device 1 is high (bit 1), the current is high enough to switch the DW in device 2, changing it from a 1 bit to a 0 bit. In Step 2, Fig. 5b, we apply a voltage pulse to CLK_2_ with CLK_3_ grounded to read device 2 while writing device 3. Since device 2's output current is now low (bit 0), the DW in device 3 does not switch and it stays as a bit 1.

Figure 5c shows an SEM image of a fabricated three inverters circuit, initialized in the inverter configuration with *μ*_0_*H*_B_=3.0 mT. In Fig. 5d, we measure *R*_MTJ_ as the information propagates around the circuit. The devices are clocked in sequence by 4.5-V pulses such that at each stage the data token is inverted. The voltage amplitude of 4.5 V is optimized for device 3, such that when device 2 is in the antiparallel state the DW of device 3 does not switch. To see the inverting operation, each resistance is normalized to its own *R*_P_. The output resistance at each pulse step oscillates between *R*_P_ and *R*_AP_ as we move around the circuit, showing oscillation between 1 and 0 bits. Operation is stopped after four steps because additional propagation requires a reset step to return all DWs to the left side. We expect that scaled down devices or materials with lower threshold currents should operate without the need of a bias field, allowing the reset to occur during reading.

**Figure 6:**
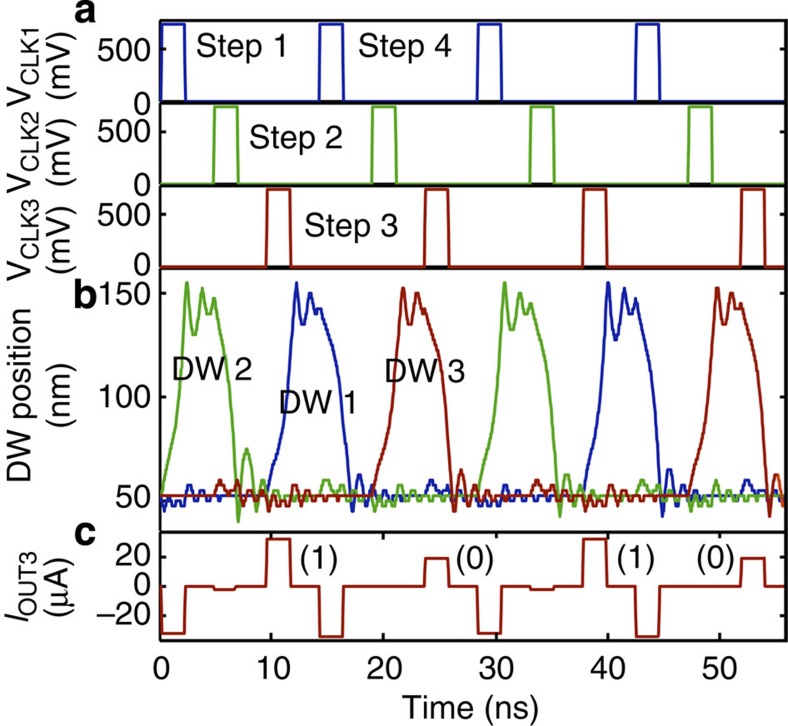
Simulation of scaled three inverters circuit. Micromagnetic/circuit simulation of transient behaviour predicted in a scaled-down device circuit with *w*=15 nm and *TMR*=100%. (**a**) 0.7 V, 2 ns clock voltages for each device applied sequentially in time. (**b**) Simulation of the domain wall (DW) behaviour of each device, where we can see the DW switches between the left side of the device (∼50 nm) and the right (∼150 nm). Once the DW switches, it oscillates about its position. (**c**), Current at the *I*_OUT3_ node from Fig. 5a versus time. The positive current oscillates between bit 1 (32 μA) and bit 0 (19 μA). The negative current is from current flowing back from subsequent clock pulses, but it does not disturb the DW position in device 3.

In Fig. 6, we model the behaviour of a scaled three-inverter circuit using a transient micromagnetic/circuit simulation^1^ to predict scaling, switching energy, and switching delay. For the micromagnetic simulation, we use the Object-Oriented Micromagnetic Framework^28^ model with a current-induced DW motion extension^29^. We assume *t*=2.5 nm, *w*=15 nm, wire length 200 nm, TMR=100%, and material properties for in-plane anisotropy CoFeB (exchange energy 13 × 10^−12^ J m^−1^, damping parameter *α*=0.01, non-adiabatic parameter *β*=0.05, saturation magnetization *M*_S_=8 × 10^5^ A m^−1^, and spin polarization factor *P*=0.4 (ref. 18)). The simulation inputs current and outputs the new DW position in the wire. We then convert the DW position to a new *R*_MTJ_ value, which we input into the three-terminal SPICE circuit model^30^ shown in Fig. 2b. The circuit model calculates *I* and *V* at every node of the circuit, which we input back into the micromagnetic model. We repeat this iterative process every 0.05 ns over the timescale of interest.

Figure 6 shows the simulated transient circuit behaviour. The information flows sequentially, driven by 0.7 V, 2-ns clock pulses. At Step 1 (equivalent to Fig. 5a) we apply *V*_CLK1_, shown by the blue voltage pulse. The DW position versus time plot shows that the DW of device 2 switches across the device (green curve). At Step 2 (equivalent to Fig. 5b) we apply *V*_CLK2_, shown by the green voltage pulse. In the DW position versus time plot, the red curve represents the position of the DW in device 3, and we see that its DW does not switch at Step 2. This oscillatory behaviour is repeated in Step 3, Step 4 and so on. The data token is inverted every stage, meaning that the DW moves in every other device, for example, device 2 (green), device 1 (blue) and device 3 (red). The simulated circuit differs from the implementation, however, because the implementation uses a bias field. In the absence of the bias field in the scaled simulation, the logic resets itself during the read operation. By monitoring the *I*_OUT_ node in the simulation, we see it oscillates between high current (bit 1, 32 μA) and low current (bit 0, 19 μA) depending on the DW position of device 3. The negative pulses in the current transient occur when current is flowing backward from the next gate.

## Discussion

We have demonstrated the capability of DW-based devices as logic gates, including inverter, buffer, and circuit operation. The DW depinning is non-linear with a sharp switching threshold *δV*<0.01 V, allowing stable 0 and 1 bits below and above the DW depinning voltage. The gates exhibit gain, one gate can drive two subsequent gates, and the three-device circuits shows that they can be cascaded into circuits. The micromagnetic/circuit simulation shows analogous switching behaviour to the experiment in further scaled devices.

While the information is encoded in the DW position, the inputs and outputs are current, maintaining compatibility with conventional field-effect transistors. The gate architecture is simple and adaptable to recent advances in magnetic materials, allowing an increase in Δ*I/I* up to 100–600% and a reduction in power consumption and *J*_T_ by 100 × . These results show that magnetic-based logic has a realistic path to implementation, and may provide the basis for digital systems at the end of the semiconductor roadmap.

## Methods

### Fabrication

Thin film growth was done using UHV d.c. magnetron sputtering^31^. The sample was annealed *in situ* at 280 °C for 30 min with MgO exposed to vacuum to crystalize the CoFeB layer, and *ex situ* at 300 °C for 1 h in a 1-tesla field to set the IrMn exchange bias. Patterning was done using hydrogen silsesquioxane (HSQ) electron-beam resist and a Raith 150 electron-beam lithography tool. The pattern was etched into the thin film using non-reactive ion beam etching. HSQ was used as a spin-on insulator to prevent shorting of the device. A further electron-beam lithography step using polymethyl methacrylate was done to open holes on top of the three terminals for electrical contact. The darker ellipse on top of the MTJ in Fig. 1c shows a hole patterned in the SiO_2_ insulating layer to electrically contact the MTJ top. Alignment of the hole on top of the MTJ to within 50 nm between two electron-beam lithography steps limited the initial size of the prototypes. Then Ta (5 nm)/Au (135 nm) electrodes were placed with an additional electron-beam lithography step.

### Resistance and current-driven measurements

For testing, the devices were wire bonded to a chip carrier and placed in an electromagnet. A signal multiplexer alternated the device terminal connections between a resistance measuring set-up and a pulse driving set-up.

In the resistance measuring set-up, a lock-in amplifier performed a four-point resistance measurement by forcing a sinusoidal voltage (100 mV, 6.25 Hz quasi-DC) between one OUT terminal and the IN terminal and sensing the output voltage between a second OUT terminal and the CLK terminal. The forced current was known and kept low by a 100 kΩ ballast resistor connected in series with the measured resistance.

In the pulse driving set-up, a pulse generator provided the applied current pulse across the DW track between the IN and CLK terminals.

## Additional information

**How to cite this article**: Currivan-Incorvia, J. A. *et al*. Logic circuit prototypes for three-terminal magnetic tunnel junctions with mobile domain walls. *Nat. Commun.* 7:10275 doi: 10.1038/ncomms10275 (2016).

## Supplementary Material

Supplementary Information Supplementary Figures 1-3 and Supplementary References.
