# Supplementary material for: Logic circuit prototypes for three-terminal magnetic tunnel junctions with mobile domain walls
Source: Nat Commun. 2016 Jan 12;7:10275. doi: 10.1038/ncomms10275 (PMC4729928; doi:10.1038/ncomms10275)
Supplement: Supplementary Information — Supplementary Figures 1-3 and Supplementary References. [file ncomms10275-s1.pdf]

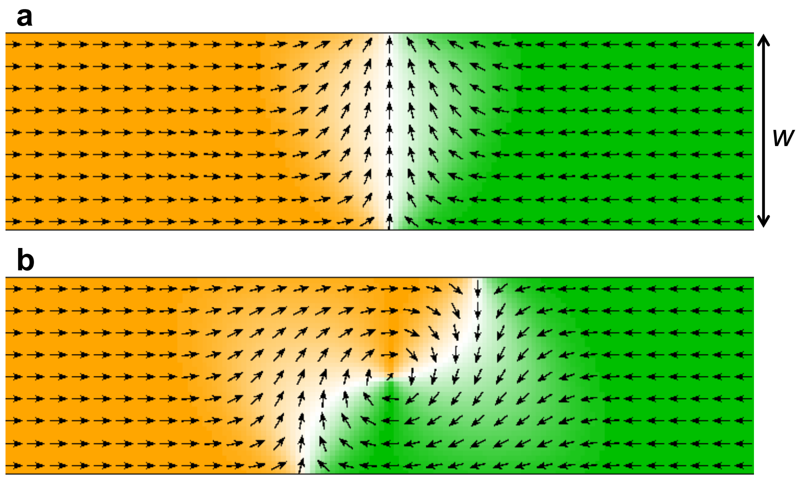

Supplementary Figure 1 | Simulation of domain wall type. a, Micromagnetic simulation of a *w* = 400 nm magnetic wire initialized with a transverse domain wall (DW). Total energy of the system is *E* = 5.17 aJ. **b**, Wire initialized instead with a vortex wall. *E* = 5.67 aJ. The DW-logic devices have wire width *w* = 400 and thickness *t* = 4 nm. The type of DW in these devices is a function of *t* and *w*: in thin, narrow wires the DW is transverse, but it transitions to a vortex shape in thicker, wider wires[1,2]. We estimate the DW type in our devices using micromagnetic modeling. We model wire sections 4 nm × 2 μm × 400 nm, with standard material parameters for CoFeB[3]: saturation magnetization $M_S = 6 \times 10^5$ A m^{-1}, exchange stiffness A = 1×10^{-11} J m^{-1}, and a small random anisotropy of 100 J m^{-3}. We initialize the simulation with either a transverse or vortex DW and allow the energy to relax. The DW type with lower final total energy of the system, *E*, is the more energy-favorable type. **a**, **b** shows a top-down micromagnetic image of the *w* = 400 nm wire initialized with a transverse DW and a vortex DW, respectively. We find $E_{transverse}$ = 5.17 aJ and E_{vortex} = 5.67 aJ. Thus, we predict that a transverse DW is the lower energy configuration. While these wires are fairly wide due to constraints in multiple alignments during fabrication, the CoFeB layer is thin enough that a transverse DW is more favorable.

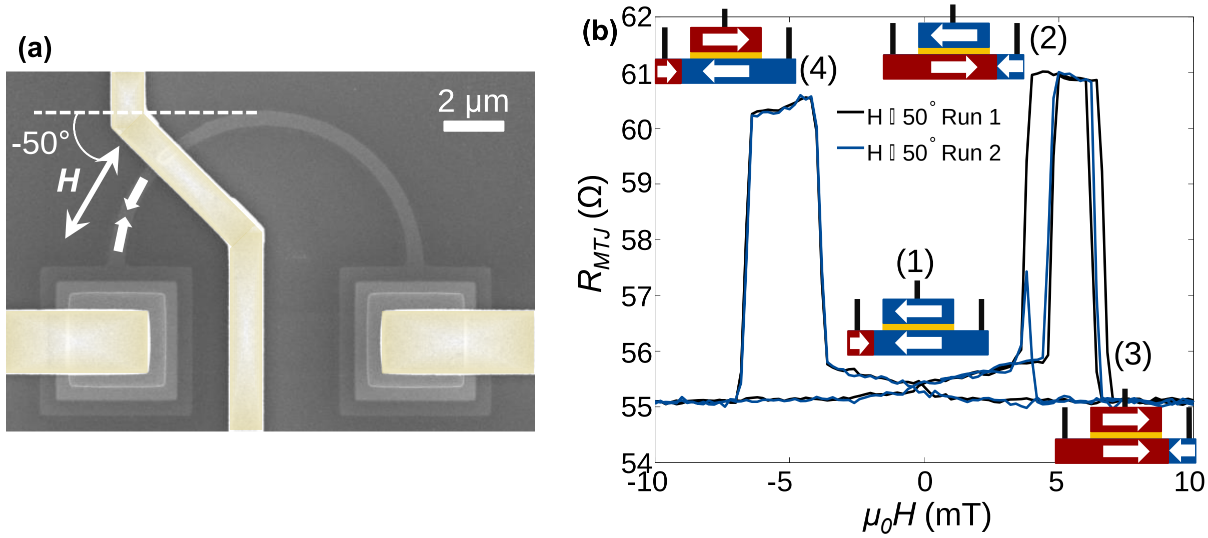

Supplementary Figure 2 | Field-driven device behavior. a, Scanning electron micrograph of a device depicting the angle of applied field *H*. We saturate the sample so that a DW is initialized on the left of the magnetic wire, shown by the white arrows. *H* is set at a -50° angle from the dotted line to be approximately parallel with the DW. **b**, Resistance of the device's magnetic tunnel junction (MTJ) vs. *H*. The initial device configuration is depicted in cartoon (1), with the DW on the left and the MTJ in a parallel, low-resistance state. We start by sweeping the field in + $\mu_0 H$ to 20 mT. The DW switches past the MTJ at $\mu_0 H_{DW}$ = 3.6 mT to configuration (2), and eventually the MTJ top also aligns with the field at $\mu_0 H_{MTJ}$ = 6.4 mT, shown by configuration (3). Then we sweep the field in –*H*. At $\mu_0 H_{DW}$ = -3.6 mT we reach configuration (4) with a high resistance, and at $\mu_0 H_{MTJ}$ = -6.4 mT both sides of the MTJ saturate in the negative direction. To operate the device without switching the MTJ top, we need to keep the field below H_{MTJ}. We can see the switching field behavior is not exactly repeatable in the +*H* direction between two runs. This is most likely because the initialization field does not always place the DW in the exact same pinning site. But, after driving the DW and MTJ in +*H*, the depinning behavior is repeatable in –*H*.

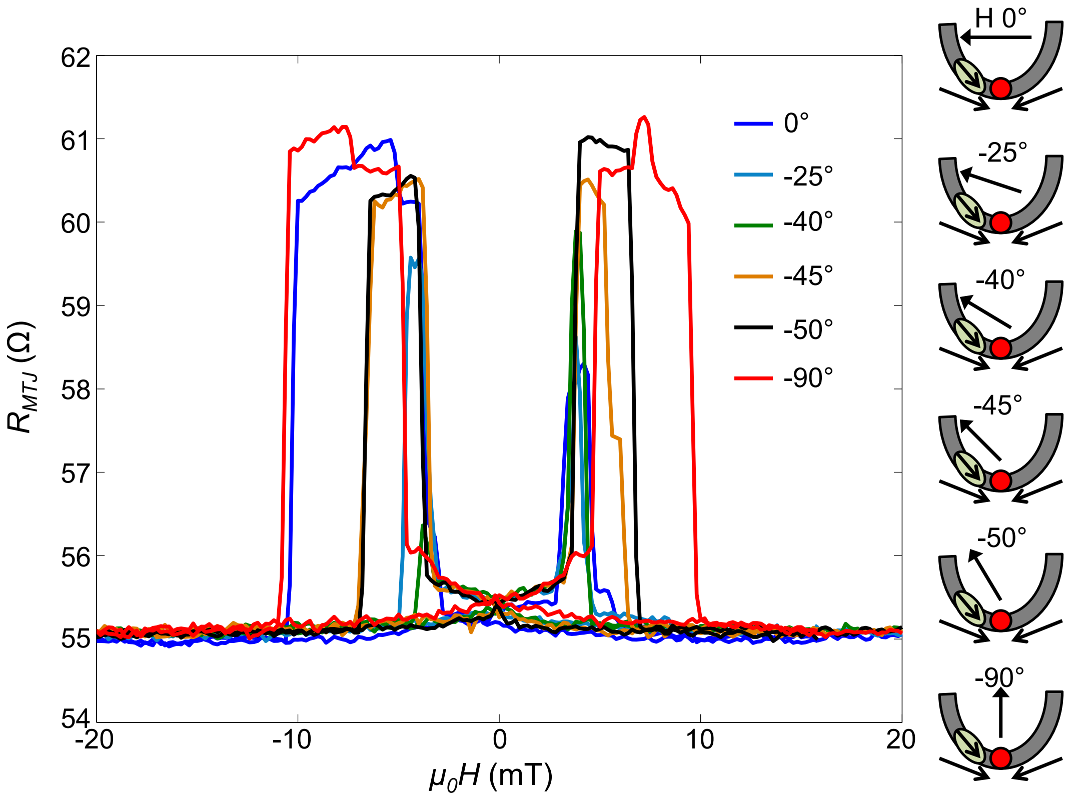

Supplementary Figure 3 | Field-driven behavior at different field angles. Plot of R_{MTJ} vs. H for six different field angles from 0° to 90°. The H_{DW} switch at ±3.6 mT is not significantly affected by the field angle. For H_{MTJ}, 0° and 90° both have the highest switch at $\mu_0 H_{\text{MTJ}}$ = ±10 mT. These angles are least aligned with the MTJ top. As we increase the field angle above 0°, H_{MTJ} increases. At -25° and -40° there is almost no spread between H_{DW} and H_{MTJ}. This is most likely because at these angles the field is more parallel to the MTJ top, so it switches at much lower fields. The cartoons show the magnetic moment direction of the magnetic wire with a DW (red dot) and the MTJ top magnet (ellipse) compared to the different field directions.

Supplementary References

1. Beach, G. S. D., Tsoi, M. and Erskine, J. L. Current-induced domain wall motion. *Journal of Magnetism and Magnetic Materials* **320,** 1272-1281 (2008).

2. Gomez, . D., Luu, . V., Pak, . O., Kirk, . J. and Chapman, . N. Domain configurations of nanostructured Permalloy elements. *Journal of Applied Physics* **85,** 6163-6165 (1999).

3. Fukami, S., Suzuki, T., Ohshima, N., Nagahara, K. and Ishiwata, N. Micromagnetic analysis of current driven domain wall motion in nanostrips with perpendicular magnetic anisotropy. *Journal of Applied Physics* **103,** 07E718 (2008).
